# Questionnaire development for the Lolland-Falster Health Study, Denmark: an iterative and incremental process

**DOI:** 10.1186/s12874-020-00931-1

**Published:** 2020-03-03

**Authors:** Cecilie Lindström Egholm, Aake Packness, Jakob Stokholm, Knud Rasmussen, Christina Ellervik, Erik Simonsen, Anne Illemann Christensen, Randi Jepsen

**Affiliations:** 1 Lolland-Falster Health Study, Centre for Epidemiological Research, Nykøbing Falster Hospital, Fjordvej 15, 4800 Nykøbing F, Denmark; 2 Data and Development Support, Region Zealand, Alléen 15, 4180 Sorø, Denmark; 3 Psychiatric Research Unit, Psychiatry, Region Zealand, Fælledvej 6, 4200 Slagelse, Denmark; 4 Research Unit of General Practice, University of Southern Denmark, J. B. Winsløws Vej 9A, 5000 Odense C, Denmark; 5 Copenhagen Prospective Studies on Asthma in Childhood, Copenhagen University Hospital Herlev-Gentofte, Ledreborg Alle 34, 2820 Gentofte, Denmark; 6 Department of Pediatrics, Slagelse Hospital, Ingemannsvej 18, 4200 Slagelse, Denmark; 7 Department of Laboratory Medicine, Boston Children’s Hospital & Harvard Medical School, Boston, MA USA; 8 Department of Clinical Medicine, Faculty of Health and Medical Sciences, University of Copenhagen, 2200 Copenhagen N, Denmark; 9 Department of Health and Morbidity in the Population, National Institute of Public Health, Studiestræde 6, 1455 Copenhagen, Denmark

**Keywords:** LOFUS, Lolland-Falster Health study, Population study, Questionnaires, Iterative and incremental process, Questionnaire development, Items, Scales, Loops of learning

## Abstract

In composing multi-thematic questionnaires for the Lolland-Falster Health Study (LOFUS), we faced a range of challenges, for which we found limited guidance in the literature. LOFUS is a household-based population study covering multiple medical and social research areas and targeting the mixed rural-provincial population of 103,000 persons on the Danish islands Lolland and Falster. Households were randomly selected for invitation. In this paper, we describe and discuss challenges in developing the questionnaires related to stakeholders, content of the questionnaire, and the process itself. The development process was characterised by loops of learning and can be described as an iterative and incremental process. We propose recommendations to researchers and administrators involved in similar development processes, including awareness of the non-linearity and complexity of the process, a need for negotiations and navigation among multiple stakeholders, and acknowledgement of pragmatism as an inherent part of decisions made in the process.

## Background

Many population studies use self-administered questionnaires for collection of data on socio-economic status, lifestyle, medical history, symptoms, quality of life, etc. These data are used for analysis of baseline associations between risk factors and reported health, and they are used for exposure classification of study participants in follow-up studies of health outcomes [1–4].

Questionnaire development for such studies seem as a straightforward task of compiling relevant and well-designed items or scales. However, the process may be considerably more challenging [5]. Methodological advice is available on how to phrase and validate single items and scales and how to design and administer a questionnaire [6–8]. Still, in composing a multi-thematic questionnaire for the general population, we faced a range of challenges related to the development process such as content, resources, stakeholders, and practical matters; issues for which we found limited guidance in the literature.

In this paper, we report lessons learned from our questionnaire development for the Lolland-Falster Health Study (LOFUS), Denmark, which included participants from households and of all ages, combined multiple medical and social research areas, and had several stakeholders [9]. The objective is to present and discuss the challenges, considerations, and trade-offs in the development process. Our experiences may help researchers in development of composite questionnaires for new population studies.

The paper is based on minutes of meeting, email correspondences, notes, and process documentation made by the first author (CLE), who was a member of the questionnaire-working group from start to finish. She drafted a thematic overview of the notes, which subsequently was discussed and organised as table and text by CLE, AP (researcher involved in a subproject), and RJ (member of the questionnaire-working group and project manager for LOFUS). The remaining authors, who were also part of the questionnaire development process as questionnaire-working group member, steering group member, principal investigator (PI), or external expert had roles as critical peers in the writing process.

### Lolland-Falster Health study (LOFUS)

LOFUS is a household-based population study, undertaken in the mixed rural-provincial population of 103,000 persons on the Danish islands Lolland and Falster aimed to form a cohort for later follow-up [9]. Data collection started in February 2016 and continued through February 2020, achieving 19,000 participants.

The aim of LOFUS was to create a research platform, including a comprehensive database and a biobank, to investigate risk factors for diseases including socio-economic, hereditary, lifestyle, familial, social, and environmental exposures on the individual, family, and household basis [9]. Randomly selected adult persons (≥18 years) from Lolland-Falster and their entire households were invited. Household members were of all ages, and they could participate together or independently. Besides questionnaires, health examinations were performed, and biological samples were collected [9].

LOFUS is owned by Region Zealand and administered by Nykøbing Falster Hospital. Region Zealand, Nykøbing Falster Hospital, and Guldborgsund and Lolland municipalities financed the project. In addition, researchers interested in collection/use of specific parts of the data contributed financially to the budget. The project is governed by a steering committee including representatives from the financing organisations, researchers with university affiliations, and administrators [9].

Lolland-Falster has a deprivation score of 44% above the Danish average based on calculation of educational level, employment rates, expenditures on social benefits, treatment of patients with mental disorders, life years lost, etc. [10, 11]. The inhabitants of Lolland-Falster report more problems with physical and mental health, unhealthy lifestyle, and lack of social contacts than inhabitants in the rest of Denmark do [12]. While LOFUS focused on a disadvantaged, rural-provincial area of Denmark [9], it was developed from the Danish General Suburban Population Study (GESUS) that included adults from a larger provincial town [13], which in turn was based on the Copenhagen City Heart Study from an area in the Copenhagen capital [14]. Basic elements of the questionnaires, health examinations, and biobank samples were similar in all studies to allow data pooling.

Early in the LOFUS planning process, local, regional, and national institutions and individual researchers were invited to submit projects to be included in LOFUS. The steering committee assessed the project-proposals on parameters such as importance, quality of the design, feasibility, and expected burden on participants. Fifteen subprojects were accepted, representing a broad spectrum of disciplines and data collection requests, all of them including questionnaire data [9]. The PIs responsible for the subprojects signed a collaboration agreement and paid a fee. An exclusive right to use data collected for a particular subproject was granted for 3 years, after which data would be available for other researchers upon application to the steering committee.

A questionnaire-working group was formed to work specifically with the scientific, administrative, and practical aspects of developing questionnaires for the study. The formal decision-making authority rested with the steering committee. Below, we describe and discuss the questionnaire development process.

#### Questionnaire development – an iterative process

Although the task of compiling a composite questionnaire for the LOFUS study initially seemed rather straightforward, it soon emerged a highly complex task, where neither the final result nor the process itself were clearly defined in advance. In hindsight, the questionnaire development can be described as an iterative and incremental process. Iterative and incremental development has been applied for decades e.g. in software design [15]. Characterized by starting with an idea rather than a clearly defined final solution, development occurs through loops of learning with constant revisions of requests and solutions. The evolving product is continuously tested and evaluated, until a satisfactory product is in place. Once there, it moves out of the iterative loops to implementation [15].

**Fig. 1:**
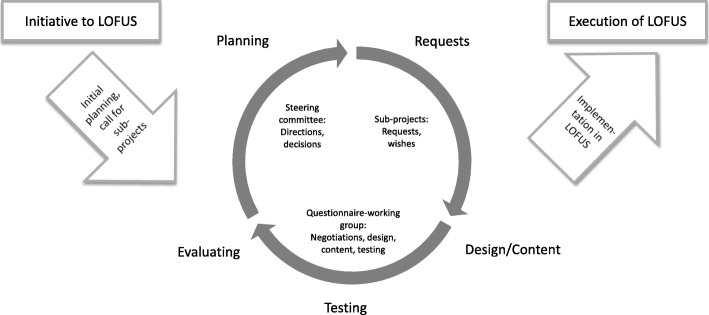
The iterative and incremental process for the development of a composite questionnaire for the Lolland-Falster Health Study (LOFUS)

In LOFUS, our starting point was a broadly defined research aim [9] and the questionnaire from the affiliated Danish General Suburban Population Study [13]. We faced a task of adapting and combining this with a new target population including children and youth and the requests from the accepted subprojects. The Danish General Suburban Population Study-questionnaire consisted of 335 items, and suggestions from the subprojects added several hundred extra items. Although these items had scientific relevance, they were too numerous to be feasible to implement. Adding to the complexity, the logistics for the entire LOFUS study (including recruitment, health examinations, collection of biological samples, and development of the research database) were under development in parallel processes, necessitating mutual adaptions along the way. Figure 1 illustrates the iterative and incremental process for the development of the composite LOFUS questionnaire, and in the following, we describe and discuss some of the main issues related to the stakeholders and the process, as well as the content of the questionnaire and practical matters.

### Stakeholders

The LOFUS questionnaire development involved a consortium of stakeholders including the questionnaire-working group, the steering committee, and several external PIs for subprojects, who had to reach agreement throughout the process’ iterations.

The questionnaire-working group consisted of two to three steering committee members and a varying number of external members. The group was marked by turnover, with only one consistent, administrative member throughout the whole process. All members had experience with questionnaire-based research; however, only a few had previously worked with composite questionnaires in population studies. During the development process, it became evident that this kind of experience benefits from being supplemented by skills in project management and negotiation, a clear mandate, and a realistic time frame.

The LOFUS steering committee included administrative representatives of the financing Region Zealand and the two municipalities, which promoted their ownership and insight in the project. They contributed with local knowledge and communicated their hopes and expectations for results and effects of the LOFUS study. However, since their background was not in research, they preferred to let others decide on questionnaire matters. The researchers in the steering group represented various clinical areas and different scientific approaches, e.g., epidemiological, developmental, biological, and psychometrics, which was intended to enrich the decision-making process. However, even very skilled researchers may lack the experience of developing composite questionnaires for public health and epidemiological purposes. Consequently, halfway through the process, the steering committee selected a subgroup of steering group members and added external experts to deal with the questionnaire development process and the decision-making.

The local municipalities were represented in the development process; however, the target population, i. e. the general population of Lolland-Falster, was not. In the years since the start of the process, Denmark has seen a momentum in involvement of patients and other target groups in research, and had the planning started now, lay representatives would have been involved [16].

The subprojects’ PIs represented many different clinical and non-clinical research areas, organisations, and academic ranks. In negotiations, we experienced that certain scientific interests could be associated with individual career interests, which was challenging to deal with especially when questionnaire requests were not met. Turning down requests could potentially affect not only the subproject per se but also career opportunities, and to some degree, such factors were taken into considerations in the steering group’s final decisions.

### The process

Working through the iterative loops (Fig. 1), the questionnaire-working group aimed to accommodate wishes from the broad range of stakeholders. Consequently, the group conducted individual negotiations with the subprojects’ PIs about inclusion of items and scales. These negotiations were challenging due to conflicting stakeholder interests, criteria for inclusion and exclusion that were under continuous development, and an unclear mandate. Consequently, the steering group meeting frequency was intensified to evaluate the recent progress and experiences, give further directions, and make decisions. In addition to the individual negotiations, all stakeholders gathered regularly to be informed about the process, to share knowledge, and to discuss possible solutions. This co-creative process contributed positively to a sense of ownership and understanding and helped reaching feasible scientific and practical solutions. Despite the challenges of negotiating, we found the iterative process to be timely and resource effective, as results of each iteration quickly could be presented to relevant stakeholders and adjusted if necessary. Had we applied a strictly linear/sequential process [15], quick series of amendments would not have been possible, potentially making it necessary to re-work an entire questionnaire at some point.

An inherent challenge with iterative and incremental processes, moving quickly from the basic idea to making the product, is that it is difficult to estimate the time required to finalise a product, which is not well defined from the beginning [15]. As mentioned above, we initially assumed the task to be straightforward, which meant that the complexity and the scope of the work were underestimated. This challenged both the working group and the steering committee since it was difficult to get expectations aligned with the actual process. While initially estimated to last 1 year, it took approximately 2 years from the time the subprojects were approved, until the composite LOFUS questionnaire was ready for implementation. Our experience indicates that acknowledging the non-linearity of the process beforehand may support the development of a realistic plan.

Another intrinsic part of iterative and incremental processes is the considerable amount of testing required to reach a satisfying final product [15]. Through pre-testing of evolving parts and versions of the questionnaire, the working group assessed comprehensibility and response burden among colleagues, families, and neighbours. Formal assessment by two groups of external questionnaire experts was conducted, providing suggestions on order of items/scales and layout. Furthermore, the pre-final questionnaire was tested on 300 responders from a random sample of the target population, who completed a paper-based version and were interviewed by telephone about the content and wording of selected items, the order of items, and the overall length of the questionnaire. Every new iteration was based on improvements identified in the past loop, until a point was reached when the questionnaire met the scientific requirements, and all stakeholders could accept the design and feasibility. Finally, LOFUS pilot-tested all procedures comprised in the full data collection, including the questionnaire. In hindsight, we realise how beneficial it has been for subsequent recap and reference that we made rigorous documentation of all revisions.

### Questionnaire content and practical matters

The questionnaire development process required many decisions about a variety of issues such as content (themes, items/scales), age-group relevance, response burden, and practical matters.

It was clear from the beginning that inclusion and exclusion of themes and items/scales in the questionnaire should be guided by scientific importance and relevance as well as validity. In addition, information already available through Danish registers [17] should not be prioritised. However, the various and changing requests that emerged during the process imposed a need to balance scientific rigour against a pragmatic approach that took into consideration intertwined factors such as fairness between subprojects, harmonisation between age-divided questionnaires, response burden, and governing organisations’ politically prioritised fields. Compromises became commonplace in the process, and often, there seemed to be no ‘right’ solutions. Table 1 presents the full list of criteria that was developed through the loops of learning and that guided the final inclusion and exclusion of themes and items/scales.

**Table 1:** Criteria guiding inclusion and exclusion of themes and items/scales in the Lolland-Falster Health Study (LOFUS) questionnaires

Criteria guiding inclusion	Explanation
Importance and relevance	Assessed by steering committee based on protocols from PIs applying for data collection in LOFUS, included aspects such as clear research question and clinical, public health, or theoretical importance.
Fairness	Balancing needs and wishes of each subproject.
Validity	Use of validated items/scales to save time and resources, allow for comparison between studies, and promote publications. Alternatively, inclusion of items/scales previously used in population surveys to allow for comparisons between studies. Otherwise, inclusion of in-house made items.
Length (feasibility)	Weighting of depth against breadth and response burden. Negotiations with subprojects on “need-to-know” versus “nice-to-know” to cut length without compromising research aims.
Acceptance/ethical considerations	Weighting of potentially negative effects of including sensitive and/or offensive items/scales against arguments for inclusion, e.g. politically prioritised fields of interest.
Simplicity/easy to understand	Weighting of simplicity and comprehensibility due to relatively high illiteracy in the target population.
Harmonization	Inclusion of items/scales that could be used across age-groups or merged with data from other studies.
Criteria guiding exclusion	Explanation
Data available in registries	Limiting response burden by using information that could be retrieved from national registries.
Diagnoses	Information about medical diagnoses are more reliable through national registries than through self-reported questionnaire.
Copyright on items/scales^a^	Copyright may limit design options of questionnaires and add extra cost.

^a^A few exceptions from this criterion were made due to special requests from subprojects

A main issue was inclusion of symptom scales normally used in clinical settings. Arguments for inclusion were wishes to assess prevalence of clinical conditions or symptoms and inform health care services. Opposing arguments were that such scales are developed as assessment tools as part of a clinical evaluation by a healthcare professional in a clinical setting, they might not validated in epidemiological studies, and the general population may regard them sensitive and/or provocative. Therefore, most suggested symptom scales were discarded.

Responders ranging from age 0 to age 100+ made it necessary to develop age-specific version of the questionnaire. We sought inspiration in other population studies and decided on four different versions for children/adolescents stratified by age 0–1, 2–3, 4–10, and 11–17 years, and one version for adults aged ≥18 years. When relevant, the same items and scales were included across age groups. Details are provided in Additional file 1: Table 1a and Table 1b. Acknowledging that age-related cognitive functioning has profound implications for the question-answer process [18], we decided on age 11 as the cut-off point of when the parents/guardians or the children themselves should fill in the questionnaire. In addition to a core questionnaire for each age-group (Additional file 1: Table 1a), additional items/scales were triggered by gender, age, or particular responses to items in the core questionnaire (Additional file 1: Table 1b). Some subprojects required a limited number of responders only, which over time allowed for replacement of some scales. The shortest age-specific core questionnaire were for participants aged 0–1 years and included 54 items, and the longest were for participants aged ≥18 years and included 299 items (Additional file 1: Table 1a).

Practical matters in the questionnaire development process related to logistics, technical solutions, and finances played a role in decisions made in the process. For instance, about 16% of adult Danes have a low level of literacy [19], and the possibility of audio-computer aided response was considered. It was rejected in the loops of learning, due to the complexity of reading out questions and response options of multi-dimensional scales [20]. Approximately 5% of persons invited to LOFUS were non-Danish citizens [21] with varying Danish language proficiency. However, it was decided that LOFUS could not prioritise resources for translation into relevant languages. Thus, only participants who mastered Danish could fill in the questionnaires.

## Conclusion

Cohort studies are expensive, and therefore it is of utmost importance to optimize the data collection tools and make them as scientifically relevant and feasible as possible. Based on our experience, meeting minutes, notes, and process documentation, we have described an iterative and incremental process development of LOFUS questionnaires and given some examples of our challenges, methods, and trade-offs. Being well aware that specific research aims and local context always will be important for the specific ‘how?’ and ‘what?’, we have attempted to extract the main lessons learned from the process, which we believe will be useful for researchers and administrators facing the task of developing a composite questionnaire for a population or cohort study.

Firstly, we recommend that questionnaire development (and indeed, all elements of a population-based study) should be approached as an iterative and incremental process. It is most likely not possible to develop a composite questionnaire step by step in a linear working process, as this requires clear criteria for the final result in terms of length, content, design, distribution etc. Continuous loops of learning, on the contrary, allow for agile working processes and progressing adjustments. The steering committee and project owners should acknowledge and consider that engagement of stakeholders – including the target population – in co-creation, inevitably will take considerable time and resources, but it will also contribute to ownership, shared problem solving, and partnership.

Secondly, we suggest that the process of developing a composite questionnaire requires a balance between scientific rigour and pragmatism. Thus, logistics, financial burden, and feasibility may impose on the scientific, methodological ideals. To prevent frustration, involved staff and stakeholders should accept this process as a prerequisite for completion of the task. The more explicitly the complexity of the questionnaire development process is articulated, the easier it may be mitigated and handled.

Thirdly, our experiences highlight that competencies in questionnaire development and validation is necessary but may not be sufficient for confident and effective management of the development of composite questionnaires in similar studies. We recommend that good project management and negotiation skills be in place.

Lastly, clear decision pathways and mandate, as well as clear inclusion and exclusion criteria for themes and items/scales are advisable to secure an effective process and avoid misunderstandings.

A limitation to the present paper is that we do not provide statistics on how the composite questionnaire performs as a whole. However, affiliated subprojects are encouraged to validate the scales from which they use data and to discuss validity in their papers. Some of this work has already been published [22–24].

We acknowledge that the experiences and recommendations in this article are based on a single project. Readers may have experienced other or different learning, which we encourage them to share in order to raise awareness and promote learning. Our recommendations should always be adapted to specific contexts and projects, but the experiences of others would surely have helped and reassured us in our process.

## Supplementary information

**Additional file 1.** “Core questionnaires and additional items/scales in the Lolland-Falster Health Study.” Supplementary tables.

## Data Availability

This paper is based on the authors’ experiences, meeting notes, email correspondences, notes, and process documentation, not publicly available.

## Abbreviations

LOFUS: Lolland-Falster Health Study
PI: Principal investigator
