# Supplementary material for: Questionnaire development for the Lolland-Falster Health Study, Denmark: an iterative and incremental process
Source: BMC Med Res Methodol. 2020 Mar 3;20:52. doi: 10.1186/s12874-020-00931-1 (PMC7055066; doi:10.1186/s12874-020-00931-1)
Supplement: Supplementary file 1 — Additional file 1. “Core questionnaires and additional items/scales in the Lolland-Falster Health Study.” Supplementary tables. [file 12874_2020_931_MOESM1_ESM.doc]

**Additional file 1.** Core questionnaires and additional items/scales in the Lolland-Falster Health Study.

Table 1a. Core questionnaires in Lolland-Falster Health Study, total number of items in brackets.

| **0-1 years** (54) | **2-3 years** (104) | **4-10 years** (111) | **11-17 years** (155) | **Adults 18+ years** (299) |
| --- | --- | --- | --- | --- |
| Family | Family | Family | Family | Health and well-being, e.g., Cantril ladder [1], WHO-5 [2] |
| Home environment | Home environment | Home environment | Friends | Mental health, e.g., MDI [3], ASS [4], PLIKS [5] |
| Day care | Day care | Day care | Bullying | Self-care |
| Breastfeeding | Breastfeeding | Diet | Diet | Diseases |
| Screen time | Diet | Physical activity | Physical activity and sport | Pain |
| Health and well-being | Sleep | Transport | Transport | Prescription and over-the-counter medication |
| Diseases and symptoms | Screen time | Sleep | Sleep | Diet |
| Ear, nose and throat problems | Health and well-being | Screen time | Screen time | Screen time |
|  | Diseases and symptoms | Health and well-being | Smoking | Physical activity and sport |
|  | Ear, nose and throat problems | Diseases and symptoms | Alcohol | Transport |
|  | Mental health and functioning, e.g., SDQ [6] | Mental health and functioning, e.g., SDQ [6] | Illegal drugs | Sleep |
|  |  |  | Health and well-being | Height and weight |
|  |  |  | Diseases and symptoms | Smoking |
|  |  |  | Mental health and functioning, e.g., SDQ [6], PLIKS [5] | Pets |
|  |  |  | Life events | Alcohol |
|  |  |  | Puberty | Illegal drugs |
|  |  |  |  | Periods, pregnancies and births^a^ (7) |
|  |  |  |  | Family and friends |
|  |  |  |  | Life events |
|  |  |  |  | Marital status |
|  |  |  |  | Education and work status |

^a^Items for female participants only.

SDQ: Strengths and Difficulties Questionnaire; MDI: Major Depression Inventory; ASS: Anxiety Symptom Scale [Danish: Angst-Symptom-Spørgeskemaet]; PLIKS: Psychosis-like symptoms.

Table 1b. Additional items/scales in the Lolland-Falster Health Study questionnaires.

| **0-1 years** | **2-3 years** | **4-10 years** | **11-17 years** | **Adults 18+ years** |
| --- | --- | --- | --- | --- |
| Otitis media^b^ | Otitis media^b^ | Ear, nose and throat problems^c^ | Family relationships^c^ | Barriers to assessing mental health care^b^ |
|  |  | Otitis media^b^ |  | Personality^c^, BFI [7] |
|  |  |  |  | Health literacy^c^, HLQ [8] |
|  |  |  |  | Workload^b,c^ |
|  |  |  |  | Ear, nose and throat problems^b,c^ |
|  |  |  |  | Tonsillitis^c,d^ |
|  |  |  |  | Snoring and apnoea^b,c^ |
|  |  |  |  | Frailty and functions^d^, SHARE-FI [9] |
|  |  |  |  | Urinary incontinence in women^e^, ICQI-UI SF and ICIQ-OAB 08/04 [10] |
|  |  |  |  | Housing and indoor environment^e^ |
|  |  |  |  | Traffic noise^c^ |
|  |  |  |  | Wind turbine noise^c^ |

^b^Triggered by response to selected items in the core questionnaire.

^e^Administrered for a defined number of responses or period of time.

^d^Triggered by age.

^e^Triggered by gender.

BFI: The Big Five Inventory; HLQ: Health Literacy Questionnaire; SHARE.FI: Survey of Health, Ageing and Retirement Frailty Instrument; ICQI-UI SF: International Consultation on Incontinence Modular Questionnaire – Urinary Incontinence Short-form; ICIQ-OAB 08/04: International Consultation on Incontinence Modular Questionnaire – Overactive Bladder.

REFERENCES

1. Cantril H. The pattern of human concerns. New Brunswick: Rutgers University Press; 1965.

2. Topp CW, Østergaard SD, Søndergaard S, Bech P. The WHO-5 well-being index: A systematic review of the literature. Psychother Psychosom. 2015;84:167–76.

3. Nielsen MG, Ørnbøl E, Bech P, Vestergaard M, Christensen KS. The criterion validity of the web-based major depression inventory when used on clinical suspicion of depression in primary care. Clin Epidemiol. 2017;9:355–65.

4. Gerlach J, Psykiatrifonden. The anxiety book. The symptoms, causes and treatment of anxiety [in Danish]. Kbh.: PsykiatriFonden; 2008.

5. Nordgaard J, Buch-Pedersen M, Hastrup LH, Haahr UH, Simonsen E. Measuring Psychotic-Like Experiences in the General Population. Psychopathology. 2019;52:240–7.

6. Goodman R. Psychometric Properties of the Strengths and Difficulties Questionnaire. J Am Acad Child Adolesc Psychiatry. 2001;40:1337–45.

7. Gosling SD, Rentfrow PJ, Swann WB. A very brief measure of the Big-Five personality domains. J Res Pers. 2003;37:504–28.

8. Osborne RH, Batterham RW, Elsworth GR, Hawkins M, Buchbinder R. The grounded psychometric development and initial validation of the Health Literacy Questionnaire (HLQ). BMC Public Health. 2013;13:658. doi:10.1186/1471-2458-13-658.

9. Jacobsen KK, Jepsen R, Lembeck MA, Nilsson C, Holm E. Associations between the SHARE frailty phenotype and common frailty characteristics: Evidence from a large Danish population study. BMJ Open. 2019;9. doi:10.1136/bmjopen-2019-032597.

10. Avery K, Donovan J, Peters TJ, Shaw C, Gotoh M, Abrams P. ICIQ: A brief and robust measure for evaluating the symptoms and impact of urinary incontinence. Neurourology and Urodynamics. 2004;23:322–30.
